# Downregulated lincRNA HOTAIR expression in ovarian cancer stem cells decreases its tumorgeniesis and metastasis by inhibiting epithelial-mesenchymal transition

**DOI:** 10.1186/s12935-015-0174-4

**Published:** 2015-02-25

**Authors:** Jing Wang, Dengyu Chen, Xiangfeng He, Yuxia Zhang, Fangfang Shi, Di Wu, Junsong Chen, Ying Zhang, Fengsu Zhao, Jun Dou

**Affiliations:** Department of Gynecology & Obstetrics, Zhongda Hospital, School of Medicine, Southeast University, Nanjing, 210009 China; Department of Pathogenic Biology and Immunology of School of Medicine, Southeast University, Nanjing, 210009 China; Bengbu Medical School, Department of Microbiology, Bengbu, 233030 China; Department of Medical Oncology, Affiliated Tumor Hospital of Nantong University, Nantong, 226361 China; Department of Oncology, Zhongda Hospital, Southeast University, Nanjing, 210009 China

**Keywords:** Epithelial ovarian cancer, Cancer stem cells, LincRNA HOTAIR, Epithelial-mesenchymal transition, RNA interference

## Abstract

**Background:**

Emerging evidence indicates that dysregulated long intervening non-coding RNA (lincRNA) HOTAIR correlates highly with tumor invasion and metastasis but a link between the high expression of HOTAIR and the metastatic cascade of cancer stem cells (CSCs) needs to be further studied. The purpose of this study was to investigate the effect of down-regulated HOTAIR expression on tumorgeniesis and metastasis of epithelial ovarian cancer (EOC) CSCs. CD117^+^CD44^+^CSCs were isolated from human EOC SKOV3 cell line by using a magnetic-activated cell sorting system, and were then transfected with the expression vector-based small hairpin RNA targeting HOTAIR; the stably transfected cells were selected for the study. Colony-forming, wound-healing, cellular metastasis and tumorigenicity assays were performed.

**Results:**

The results demonstrated that the HOTAIR expression in clinical EOC tissues and SKOV3 CD117^+^CD44^+^CSCs was higher than in SKOV3 tumor tissues and non-CD117^+^CD44^+^CSCs. The CD117^+^CD44^+^-shHOTAIR showed an inhibited HOTAIR expression, reduced cell migration and invasion than CD117^+^CD44^+^- scramble, suggesting the inhibition of an epithelial-mesenchymal transition. Moreover, the downregulated HOTAIR expression in CD117^+^CD44^+^ CSCs significantly decreased the tumor growth and lung metastasis in xenograft mice.

**Conclusion:**

Our findings demonstrated the shHOTAIR-mediated down-regulation of the HOTAIR expression in CD117^+^CD44^+^ CSCs can be a promising new opportunity for future clinical trials.

## Introduction

Human epithelial ovarian cancer (EOC) is one of malignant tumors in gynecological cancers and currently remains to be the number one the first leading cause of cancer-related deaths in women due to factors such as failure for its early dectection and diagnosis, its proneness to pelvic and peritoneal metastasis, and its resistance to chemotherapy after recurrence. Although many studies have been published in the past decade and have contributed to the advance of the knowledge in this field, the complex biology of EOC is still insufficiently understood [1,2]. Therefore, more recent studies have focused on the molecular mechanisms underlying the EOC progression and the new strategies for early clinical diagnosis and effective therapy [3,4].

Recent studies have demonstrated that cancer stem cells (CSCs) are responsible for tumour-initiating potential, metastasis and eventual relapse. Thus, the treatments that target CSCs may lead to the discovery of effective methods to eradicate the malignant tumor cells [5-7].

Emerging evidence supports that long intervening non-coding RNAs (lincRNAs) play a critical role in regulating cellular processes such as differentiation, proliferation, and metastasis [8,9]. *HOX transcript antisense RNA* (termed HOTAIR), one of lincRNAs, functions in epigenetic regulatory processes, interacts with polycomb repressive complex 2 and is required for histone H3 lysine-27 trimethylation of the *HOD* locus. In addtion, HOTAIR has been strongly associated with the invasion and metastasis of cancer cells [10]. Dysregulation of lncRNA HOTAIR has been considered a primary feature of several human cancers including breast cancer [10,11], hepatocellular carcinoma [12,13], colorectal cancer [14], pancreatic carcinomas [15], gastrointestinal stromal tumors [16], and human EOC [17,18]. Of the many functions of HOTAIR, as tumor regulatory factors, the one for silencing HOTAIR transcription in CSCs has remained insufficiently understood [17,19]. For this reason, we investigated whether the downregulated HOTAIR expression would decrease the human EOC SKOV3 CD117^+^CD44^+^CSC metastasis by inhibiting epithelial- mesenchymal transition (EMT) in vitro*,* as well as cellular tumorigenicity in nude mice. The data from our current study showed that epigenetic silencing of lncRNA HOTAIR in SKOV3 CD117^+^CD44^+^CSCs resulted in reduced cellular tumorgeniesis and metastasis in mouse model. This fingings suggested that the streatgy of down-regulating the HOTAIR expression may serve as a potential anti-cancer regimen for inhibiting EOC CSC’s invasiveness and metastasis. Future investigations of this possibility are fully warranted.

## Materials and methods

### Cell line

SKOV3 cell line was acquired from an ovarian cancer patient, which is a well-established ovarian cancer model system; the line was purchased from the Cellular Institute in Shanghai, China. Cells were cultured in complete media consisting of RPMI 1640, 2 mM L-glutamine, 100 U/ml penicillin, 100 μg/ml streptomycin, and 10% fetal bovine serum. The medium was refreshed every 3 days to maintain adherent cells. When the SKOV3 cells reached 90% confluence, cells were harvested with 0.25% trypsin −1 mM EDTA (Sigma-Aldrich, St. Louis, MO, USA) treatment for 2 min.

### Isolation of CD44^+^CD117^+^cells and identification of cell phenotype

CD44^+^CD117^+^cells were sorted from the SKOV-3 cell line by using the magnetic-activated cell sorting (MACS, Miltenyi Biotec., Bergisch Gladbach, Germany). First, CD44^+^subsets were isolated by using the mouse antihuman CD44 antibody coupled to magnetic microbeads (code number:130-095-194, antibody dilution,1:20, Miltenyi Biotec., Bergisch Gladbach, Germany) and followed by the magnetic column selection or depletion. Second, the resulting cells were then depleted of CD117^−^subsets by using mouse antihuman CD117 antibody coupled to magnetic microbeads (code number:130-091-332, antibody dilution,1:20, Miltenyi Biotec., Bergisch Gladbach, Germany), and we named the CD44^+^CD117^+^cells for the EOC cancer stem cells as ‘EOC SKOV-3 CD44^+^CD117^+^CSCs’ [20-22]. The isolated cells were placed in stem cell culture medium by resuspension in serum-free DMEM/F12 supplemented with 5 μg/mL insulin (Sigma-Aldrich, Missouri, USA), 20 ng/mL human recombinant epidermal growth factor (Invitrogen, CA, USA), 10 ng/mL basic fibroblast growth factor (Invitrogen, CA, USA) and 0.5% bovine serum albumin (Sigma-Aldrich, Missouri, USA) [23,24]. The isolated CD44^+^CD117^+^cells were further identified by using flow cytometer (FCM, BD, USA) [25].

### The short hairpin RNA sequence design

A short hairpin RNA sequence of lncRNA HOTAIR was designed based on the HOTAIR RNA sequence (Gene ID: 100124700) by using the siDESIGN design software (Dharmacon, http://www. thermoscientificbio.com/design-center/) and the Block-iTTM RNAi Designer (Invitrogen, Grand island, NY) as well as BLAST (http:// www. ncbi. nlm.nih.gov/BLAST). The target sequence site for HOTAIR shRNA includes 19 base pairs of the HOTAIR RNA sequence. In addition, one scramble sequence was designed as a negative control. The shRNA sequences are as follows: pSUPER-EGFP1-HOTAIR-shRNA (pSUPER- EGFP1-shHOTAIR), Forward 5′-GATCCCCGAACGGGAGTACAGAGAGATTCAAGAG A TCTCTC TGTACTCCCGTTCTTTTTGGAAA-3′; antisense,5′-AGCTTTTCCAAAAAGAACGGG A GTACAGAGAGATCTCTTGAATCTCTCTGTACTCCCGTTCGGG-3′;scramble-siRNA: sense, 5′- GATCCCCTTC TCCGAACGTGTCACGTTTCAAGAGAACGTGACACGTTCGGAGA ATTTTTGG A AA-3′; antisense, 5′-AGCTTTTCCAAAATTCTCCGAACGTGTCACGT-TCTCTTGAAACGTGAC ACGTTCGGAGAAGGG-3′. All the primers were synthesized by Gene and Technology of China in Shanghai [10].

### Construction of pSUPER-EGFP1-HOTAIR -shRNA and production of stably transfected clones

A pSUPER-EGFP1 (enhanced green fluorescent protein 1) vector was used to construct recombinant. The recombinant pSUPER-EGFP1-HOTAIR-shRNA (shHOTAIR) was developed as previously describled [10,26]. A pSUPER-EGFP1-scrambled shRNA (Scramble-HOTAIR) was used as a negative control. These recombinants were verified by the analysis of endonuclease digestion and sequencing. The shHOTAIR and SCHOTAIR were respectively transfected into CD44^+^CD117^+^CSCs and the stably transfected clones were selected with G418 (Clontech, CA). ShHOTAIR-expressing and Scramble-HOTAIR-expressing clones were labeled ‘CD44^+^CD117^+^ shHOTAIR’and ‘CD44^+^CD117^+^ scramble’, respectively.

### Clinical samples

Four fresh surgical tissue samples of EOC patients with a median age of 55 years (age range 45–63) were collected at the Department of Gynecology & Obstetrics, Zhongda Hospital, Medical School, Southeast University between March 2012 and October 2012. The four surgical tumor samples used in this study (designated T1-T5) were categorized as malignant Fesddration Internationale des Gynaecologistes et Obstetristes (FIGO) stage II-III serous adenocarcinomas. All samples were stored immediately in liquid nitrogen until analysis [21].

### Quantitative RT-PCR of HOTAIR

Total RNA was extracted from the SKOV-3 CD44^+^CD117^+^CSCs or SKOV-3 cells or microdissected tumor tissue samples of EOC patients by using a Qiagen RNeasy Kit (Qiagen, Valencia, CA, USA) following by the manufacturer’s protocol. A single-stranded cDNA was prepared by the using SuperScript III reverse transcriptase (Invitrogen). Quantitative real-time reverse transcription-PCR (qRT-PCR) was carried out on an ABI step one plus real-time system (Applied Biosystems, USA). The cDNAs were amplified by PCR with primers as follows: HOTAIR: sense, 5′-GGTAGAAAAAGCAACCACG AAGC-3′; antisense, 5′-TTGGGGAAGCA TTTT CTGAC-3′; β-actin (sense, 5′-GGACTTCGAG CAAG AGATGG-3′; antisense, 5′-AGCACTGT GTTGGCGTACAG-3′). U6-RT Primer, 5′-GTCGTATCCAG TGCAGGGTCCGAGGTATTCGCACTGGATACGACAAATATGGAAC-3′; sense, 5′-TGCGGGTGCT CGCTTCGG CAGC-3′; URP (Universal Reverse Primer), 5′-CCGGCAGGGTCCGAGGT-3′; E-cadherin: sense, 5′-TACACTGCCCAGGAGCCAGA-3′; antisense, 5′-TGGCACCAGTGTCCGGATTA-3′; Vimentin: sense, GGAACAGCATGTCCAAATCG; antisense, GCACCT GTCTCCGGTACTCA. The mRNA levels of the genes of interest were expressed as the ratio of each gene of interest to β-actin or U6 mRNA for each sample. The comparative Ct (ΔΔCt) method was used to determine the expression fold change [27,28].

### Colony forming assay

The colony formation ability of SKOV3 CD117^+^CD44^+^-shHOTAIR was investigated. A colony with a diameter larger than 75 μm or having more than 50 cells was counted for 1 positive colony according to our previous report [20]. The plate clone formation efficiency was calculated as (number of colony/number of cells inoculated) × 100%.

### Cell migration assay

To determine the role of down-regulated HOTAIR expression on migration, SKOV3 CD117^+^CD44^+^- shHOTAIR were used in the wound healing assay. Briefly, SKOV3 CD117^+^CD44^+^-shHOTAIR or SKOV3CD117^+^CD44^+^-scramble were plated in 6-well plates (5 × 10^5^ cells per well) to form a monolayer one day before the assay; non-adherent cells were removed by PBS washing. On the following day, a uniform scratch was made down in the center of the well using a sterile micropipette tip. The distance travelled by the cells was measured between the two boundaries of the acellular area at 0, 24, 48 and 96 hours respectively, after incubation. Each experiment was performed in triplicate [29].

### Cell invasion assay

The invasion ability of SKOV3 CD117^+^CD44^+^-shHOTAIR or SKOV3 CD117^+^CD44^+^-scramble was evaluated by using the transwell invasion assay as previously described [30]. Briefly, the transwell inserts with 8 μm pores were coated with Matrigel (20 μg/well; Becton Dickinson, Waltham, MA, USA); cells were seeded in the upper chamber in RPMI1640 medium supplemented with 10% fetal bovine serum. After incubation at 37°C, the cells that invaded to the lower surface of the Matrigel-coated membranes were fixed with 70% ethanol and stained with trypan blue; the cells from five randomly selected fields were then counted under a light microscope.

### Western blotting analysis

Approximately 1 × 10^6^ SKOV3 CD117^+^CD44^+^-shHOTAIR or SKOV3CD117^+^CD44^+^-scramble were collected and lyzed in the protein extraction buffer (Novagen, Madison, WI, USA), and 12% sodium dodecyl sulfate-polyacrylamide gel electrophoresis was performed and proteins (10 μg/lane) were loaded by following the pulished papers [6,31]. The rabbit antibody specific to human E-cadherin (code number: 31955) or Vimentin (code number: 57415) was used in the assay (Bioworld Technology, Dublin, OH, USA). The antibody dilution was 1: 1000.

### Tumorigenicity of shHOTAIR-SKOV3 CD117^+^CD44^+^ CSCs in an xenograft mice

Balb/c nude mice (female, weight: 16-18 g and age between 5 and 6 weeks) were ordered from the Animal Center of Yang Zhou University of China and were raised under sterile conditions at the Experimental Animal Center, Medical School of Southeast University. The experiments were performed in compliance with the guidelines of the Animal Research Ethics Board of Southeast University, China. Twelve nude mice were randomly divided into two groups of equal size (six per group): the SKOV3 CD117^+^CD44^+^- shHOTAIR group, and the SKOV3CD117^+^CD44^+^-scramble group. The nude mice was subcutaneously injected in the back with 5 × 10^4^ SKOV3 CD117^+^CD44^+^-shHOTAIR or SKOV3 CD117^+^CD44^+^-scramble. Tumor formations in each mouse were monitored every three days by taking 2-dimensional measurements of individual tumors from each mouse [32].

### Lung histopathology

Lung tissues were removed from the xenograft mice, fixed in 10% formalin, and then embedded in paraffin. Lung tissue sections of 4μm thin were cut and mounted on SuperFrost Plus glass slides; the tissues were fixed in methanol and stained in hematoxylin and eosin (HE). The slides were viewed under a Zeiss Axioplan light microscope at a magnification of × 200 [33].

### Statistical analysis

Values of interest were presented as the mean plus or minus two standard deviation. Statistical comparisons were performed using the Student’s *t*-test method. Results for all analyses with a *P* value < 0.05 indicate the statistically significant differences.

## Results

### HOTAIR expression in EOC tissues and SKOV3 CD117^+^CD44^+^ CSCs

**Figure 1:**
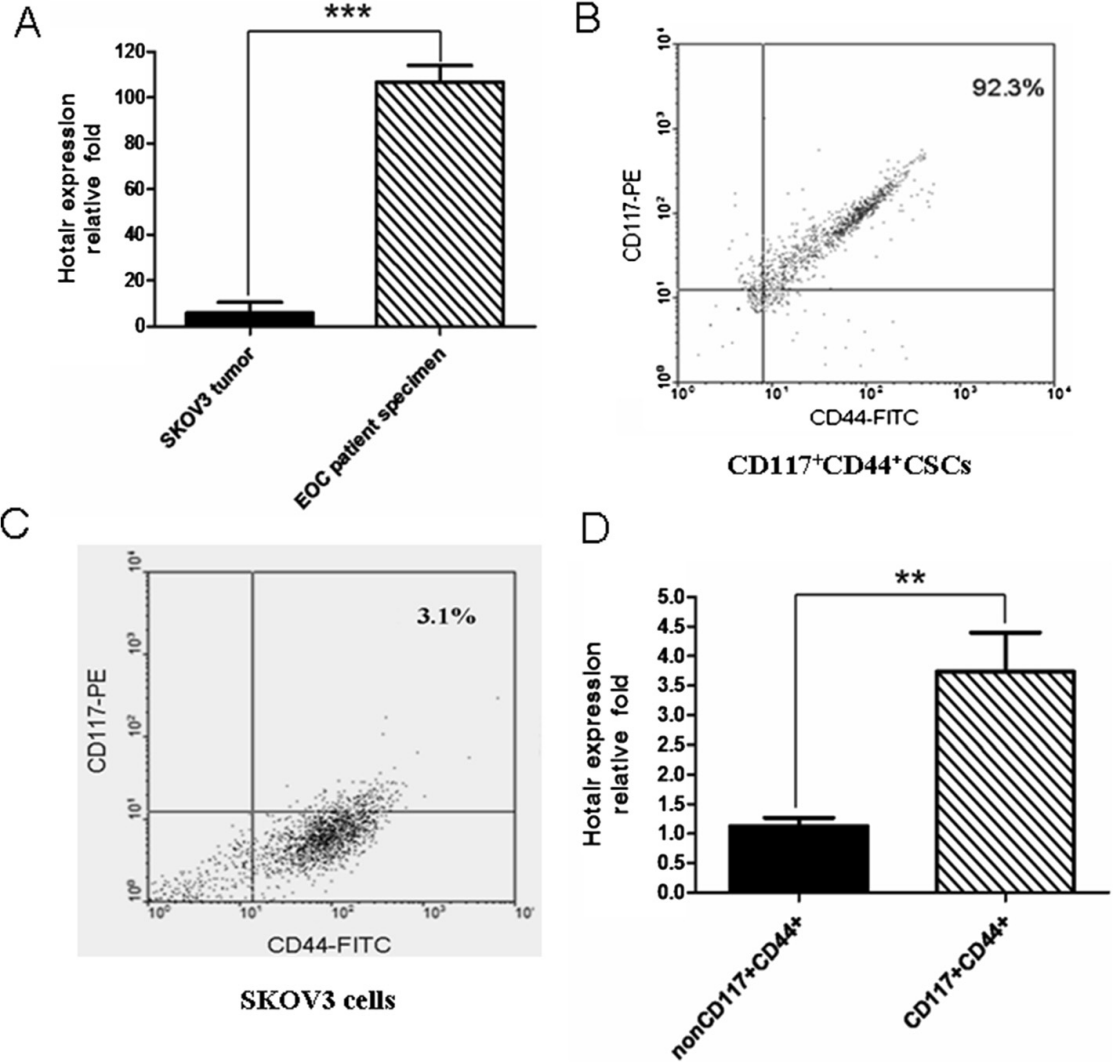
**Detection of HOTAIR expression in tumor tissues and CD117** ^**+**^ **CD44** ^**+**^ **CSCs identified by qRT-PCR and FCM. A.** The HOTAIR expression in four patient’s EOC tissues and six SKOV3 tumor tissues in the mice tested by qRT-PCR. **B** and **C**. The cell phenotype of CD44^+^CD117^+^cells sorted from the SKOV-3 cells by using the magnetic-activated cell sorting system **(B)** and the SKOV-3 cells **(C)** were analyzed by FCM, indicating 92.3% and 3.1%, respectively, of the double positive phenotypes of CD44 and CD117 in sorted cells and SKOV-3 cells. **D.** QRT-PCR analysis of the HOTAIR expression in both the CD117^+^CD44^+^ cells and the non CD117^+^CD44^+^ cells. **p < 0.01 and ***p < 0.001 were calculated by using the Student’s *t* test method, referring to the differences as indicated.

In this study, we first wanted to know whether the HOTAIR expression existed in human EOC tissues and in tumor bearing mice injected with SKOV3 cells. The result of qRT-PCR in Figure 1A shows that the HOTAIR expression not only existed in the EOC patient’s tumor tissues but its expression was significantly increased compared with SKOV3 tumor tissues in nude mice (*p <* 0.001). Next, we further investigated whether the HOTAIR expression was increased in SKOV3 CD117^+^CD44^+^CSCs compared with SKOV3 non-CD117^+^ CD44^+^CSCs in order to find a novel therapeutic target for theatment of EOC. As Figure 1B portrays, the CD117^+^CD44^+^cells, termed CD117^+^CD44^+^CSCs as described in our previous reports [6,22], were isolated by MACS, validated by FCM, and the purity of CD117^+^CD44^+^cells reached 92.3%, but there were only 3.1% of CD117^+^CD44^+^cells in SKOV3 cells (Figure 1C). Figure 1D shows the high expression of HOTAIR in CD117^+^CD44^+^CSCs in contrast with non-CD117^+^ CD44^+^ CSCs (*p <* 0.01).

### Effects of down-regulated HOTAIR in SKOV3 cells on the ability of migration and invasion

**Figure 2:**
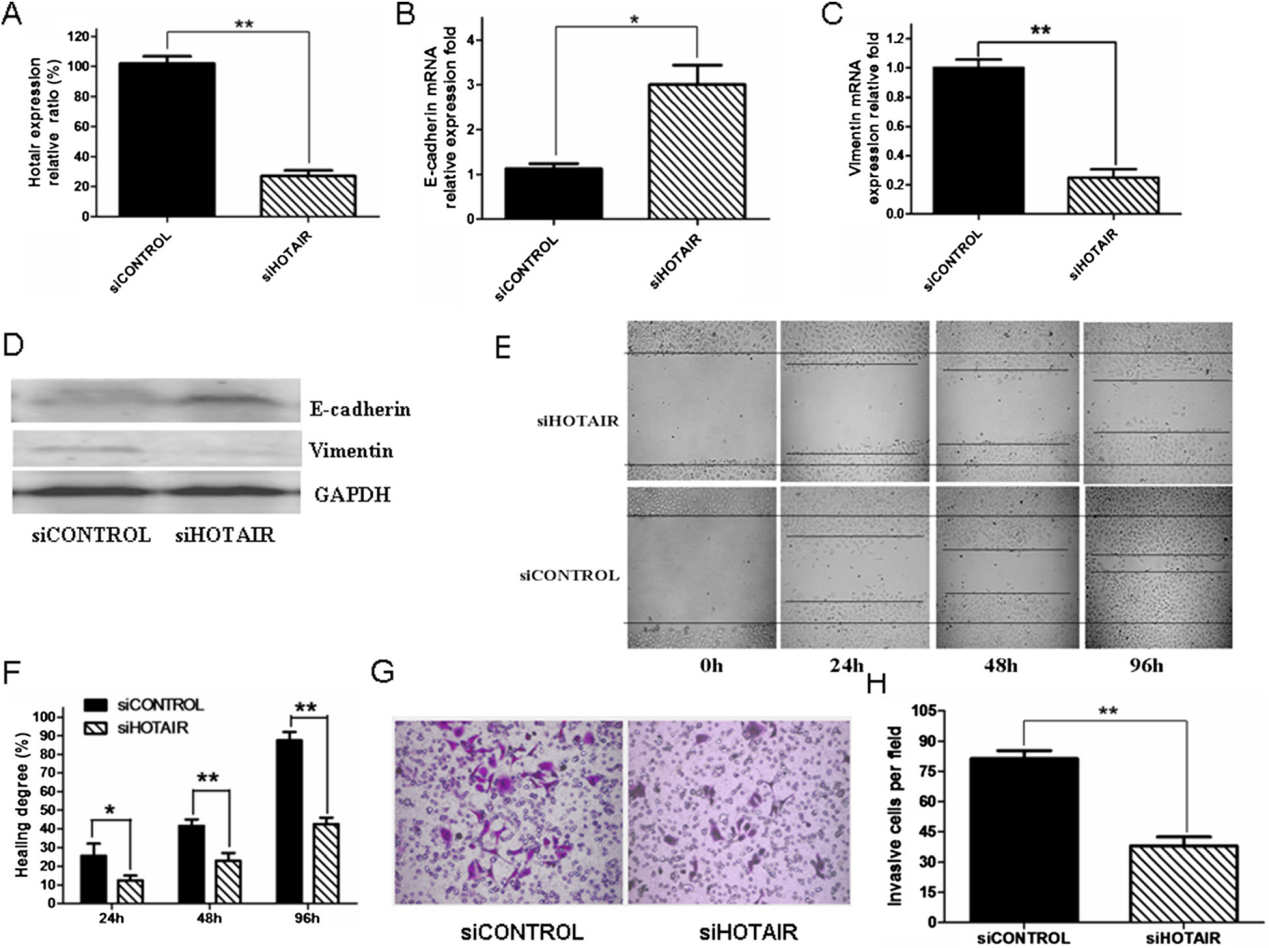
**HOTAIR**-**shRNA SKOV3 cells decreased the ability of cell migration and invasion. A-D.** RNA and proteion extracts from 1 × 10^6^ SKOV3 cells were respectively subjected to qRT-PCR and Western blot assays for detection of the expression of HOTAIR **(A)** E-cadherin **(B, D)** and Vimentin **(C, D)** in the SKOV3 cells transfected with siHOTAIR or siCONTAL (scramble). **E.** In vitro wound healing, 5 × 10^5^ different cells were respectively plated in 6-well plates to form a monolayer, and on the following day, a uniform scratch was made down in the center of well using a sterile micropipette tip. Wound closure (light microscopy, ×200) is presented the percentage reduction of the freshly wounded area. **F.** Quantification of the wound healing assay result. **G.** The invasive assay result shows that 5 × 10^5^ different SKOV3 cells were seeded in the upper chamber in RPMI1640 with serum-free. Cells that invaded to the lower surface of the Matrigel-coated membranes after being incubatied for 48 h, next fixed with 70% ethanol, and finally stained with trypan blue. Cells from five randomly selected fields were counted under a light microscope (magnification ×100). **H.** Quantification of the invasive assay result. * *P* < 0.05 and **P < 0.01, referring to the differences as indicated.

Since HOTAIR plays important regulatory roles in the malignant tumor progression through regulating cell cycle, apoptosis, invasion and metastasis, a high expression of HOTAIR correlates highly with some epithelial tumor metastasis and invasion [10,11]. Therefore, we investigated whether this correlation was changed when the HOTAIR expression was down-regulated in the SKOV3 cells. According to the previous report [10], we synthesized siHOTAIR 5′-GAACGGGAGUACAGA GAGAUU-3′ that was transfected into the SKOV3 cells. Indeed, the HOTAIR expression was markedly decreased compared with the SKOV3 cells (*p <* 0.05) 24 hours after the cells were transfected with siHOTAIR 50 nanomolar (Figure 2A). This efficacy led to the observations that the E-cadherin expression (epithelium features) was obviously increased and the Vimentin expression (mesenchymal features) was markedly decreased (Figure 2B-D). In addition, Figure 2E and G show that the migration and invasion ability of the SKOV3 cells was also remarkablely decreased compared with the SKOV3 cells transfected with scramble-HOTAIR, as is shown in Figure 2E-H (*p <* 0.05 or *p <* 0.01).

### Effect of down-regulated HOTAIR in CD117^+^CD44^+^ CSCs on the ability of colony, migration and invasion

**Figure 3:**
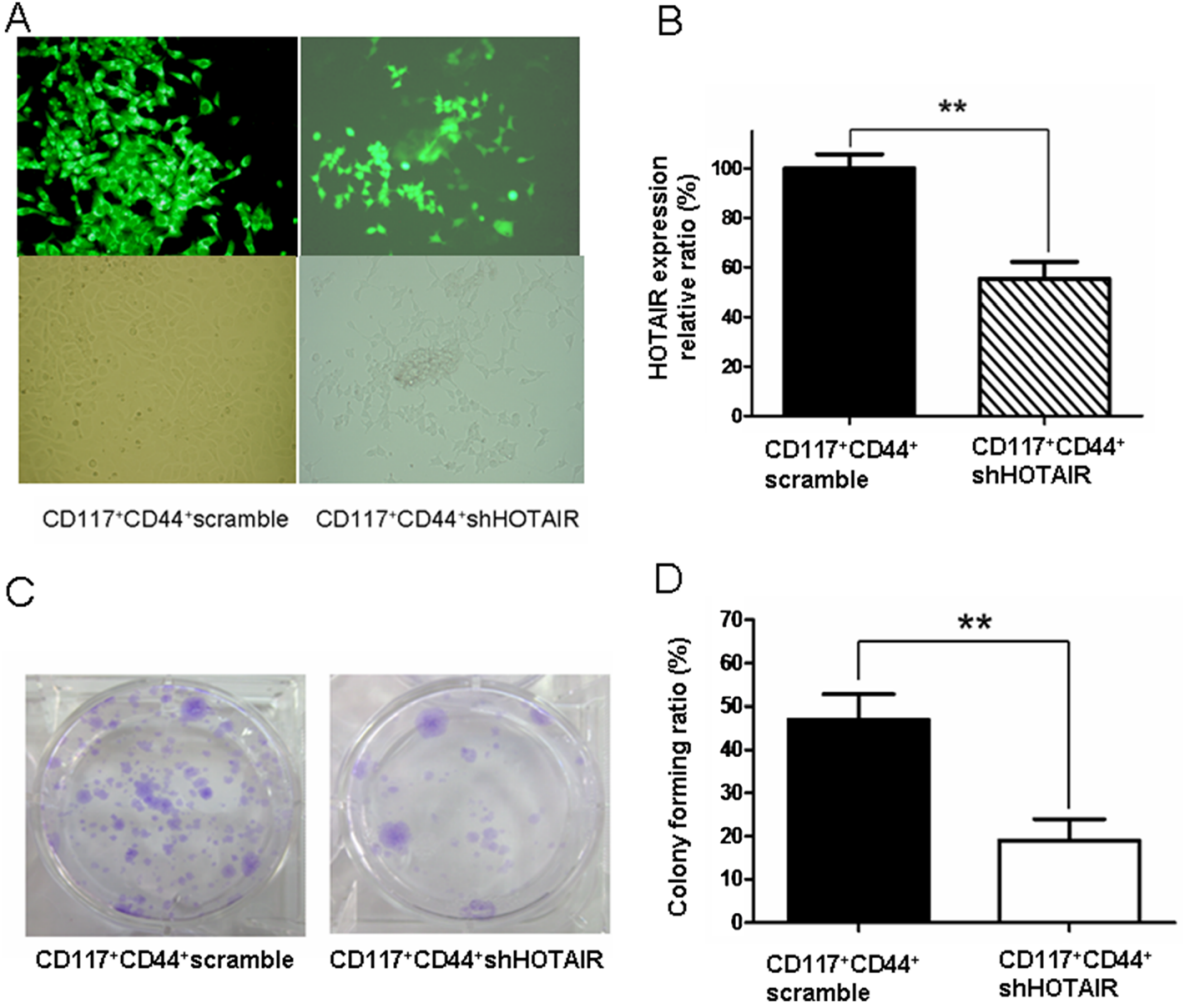
**CD117** ^+^ **CD44** ^+^-**shHOTAIR reduced the HOTAIR expression and colony forming potential. A.** CD117^+^CD44^+^ CSCs transfected with the recombinant shHOTAIR or scramble HOTAIR were selected by 800 μg/ml G 418 in two weeks (magnification 200×). Images were taken from a fluorescence microscope (top) and from a light microscope (bottom). **B.** QRT-PCR analysis of the HOTAIR expression in both CD117^+^CD44^+^-shHOTAIR and CD117^+^CD44^+^-scramble. **C.** Images of CD117^+^CD44^+^- shHOTAIR and CD117^+^CD44^+^-scramble in colony forming assay. **D.** Statistical analysis of colony forming ratio. **P < 0.01.

**Figure 4:**
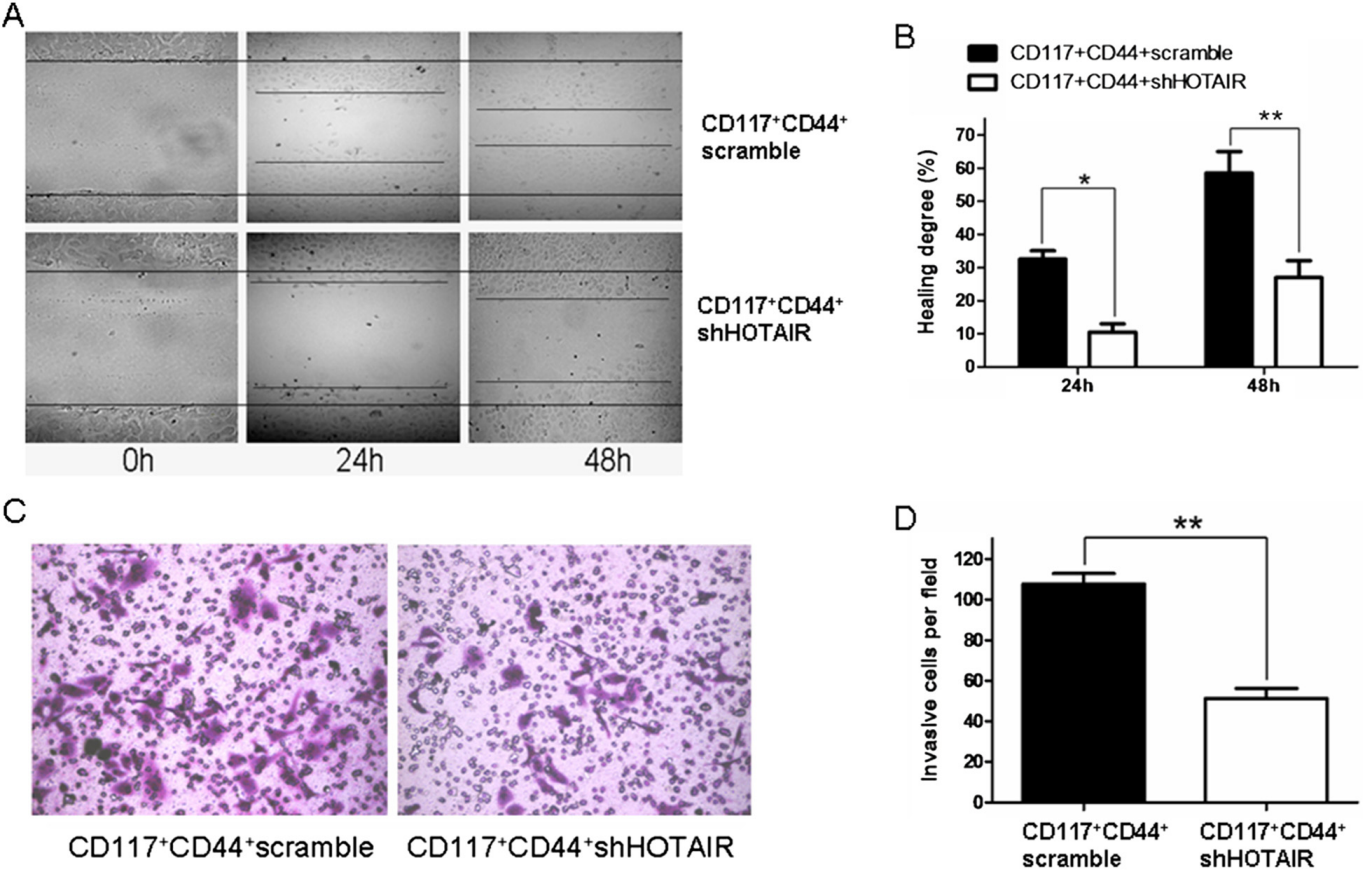
**CD117** ^+^ **CD44** ^+^-**shHOTAIR reduced its migration and invasion potential in vitro** ***.*** Images of cell migration assay results **(A)** and cell invasion assay result **(C)**. The methods are described as in Figure 2. **B.** Quantification of the metastatic assay results. **D.** Quantification of the invasive assay result. * *P* < 0.05 and **P < 0.01, referring to the differences as indicated.

To stably reduce the HOTAIR expression, we next developed the recombinant shHOTAIR to investigate the effect of down-regulated HOTAIR expression on the ability of colony, migration and invasion in SKOV3 CD117^+^CD44^+^ CSCs in vitro. The validated CD117^+^CD44^+^CSCs by FCM were transfected with the recombinant shHOTAIR (Figure 3A, right-panel) or scrambled HOTAIR (Figure 3A, left-panel), which were observed under a fluorescence microscope (top-panel) and under a light microscope (bottom-panel). The stably transfected CD117^+^CD44^+^-shHOTAIR decreased the HOTAIR expression by 54 ± 5% (Figure 3B) and reduced its colony forming rate (18 ± 6%) compared with the CD117^+^CD44^+^ scramble (46 ± 7%, Figure 3C); the differences were statistically significant (*p* < 0.01, Figure 3B and D). Figure 4A exhibits the image of the cell migration results tested by the wound healing assay. Cell migration rates at 24 and 48 hours showed a statistically significant reduction in CD117^+^CD44^+^-shHOTAIR compared with CD117^+^CD44^+^-scramble in the wound closures, particularly at 48 hours (*p* < 0.01, Figure 4A and B). In addtion, the effect of silencing HOTAIR on the invasive ability of CD117^+^CD44^+^-shHOTAIR was further evaluated by the transwell invasive assay. Figure 4C indicats the representative images of the cell invasion, which showed a marked decreased in CD117^+^CD44^+^-shHOTAIR compared with those transfected with CD117^+^CD44^+^-scramble (Figure 4D, *p <* 0.01).

### Down-regulated HOTAIR in CD117^+^CD44^+^CSCs inhibited the tumor growth and metastasis in the xenograft mouse model

**Figure 5:**
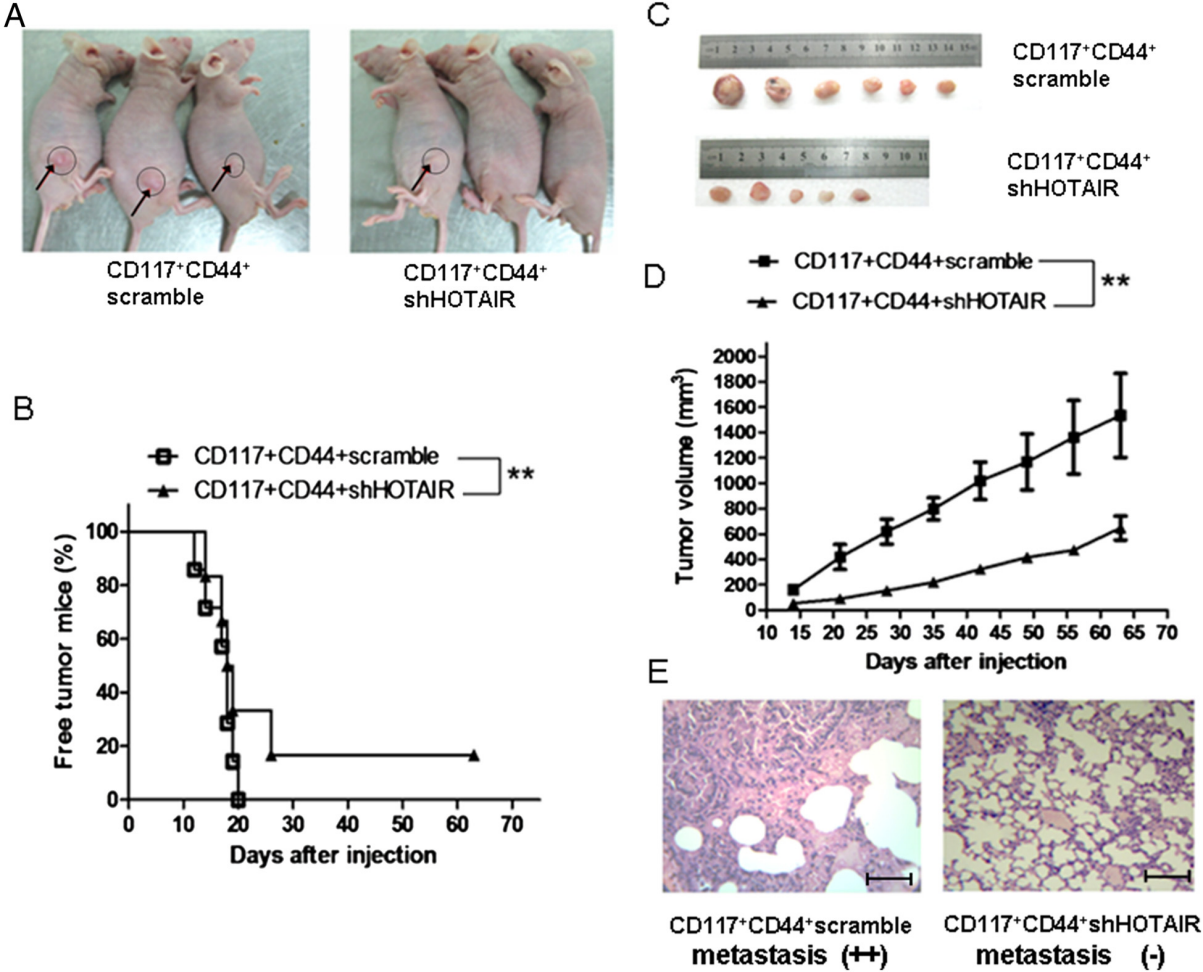
**CD117** ^**+**^ **CD44** ^**+**^-**shHOTAIR inhibited cell growth and lung metastasis in mice** ***.*** **A.** Images represent the tumor growth in the nude mice 24 days after they were injected with 2 × 10^4^ CD117^+^CD44^+^-shHOTAIR or CD117^+^CD44^+^-scramble. **B.** Tumor-free mice throughout the 64-day observation period. **C.** Tumor sizes dissected from the SKOV3 tumor bearing mice 64 days after the mice were injected with the 2 × 10^4^ CD117^+^CD44^+^-shHOTAIR or CD117^+^CD44^+^-scramble cells. **D.** Tumor volume in a set of 6 mice. **E.** Presence of tumor cell metastasis in lungs is visible in the CD117^+^CD44^+^-scramble lung tissue section (left); no tumor cells are found in the lungs of the nude mice injected with the CD117^+^CD44^+^-shHOTAIR lung tissue section (right). The sections were stained by H.E. Scale bars: 100 μm (magnification ×200). **P <0.01, referring to the differences as indicated.

After observing the effects of the down-regulated HOTAIR expression on the migration and invasion of SKOV3 CD117^+^CD44^+^-shHOTAIR in vitro, we further tested whether this effect would alter the tumorigenicity and metastatic potential of CD117^+^CD44^+^-shHOTAIR in the Balb/c nude mice. Figure 5A shows the representative images on day 24 after implantation; all the 6 mice generated tumors in 20 days after being injected with 5 × 10^4^ SKOV3 CD117^+^CD44^+^-scramble. The injected mice developed a visible tumor on Day 12, Day 14, Day 16, Day 18, Day 19, and Day 20, respectively. In contrast, 5 of the 6 mice injected with the 5 × 10^4^ SKOV3 CD117^+^CD44^+^-shHOTAIR developed visible tumors on Day 14, Day 16, Day 18, Day 26, and Day 26, respectively, and the remaining 1 mouse did not develop tumor throughout the 64-day observation period. Figure 5B presents the percentages of the tumor-free mice in the two groups. Figure 5C exhibits the images of the tumor size and quantity. Tumor growth was significantly inhibited in the mice injected with the CD117^+^CD44^+^-shHOTAIR compared with the mice injected with the CD117^+^CD44^+^-scramble, and the tumor volume was significantly reduced in the former (*p*<0.01) (Figure 5D).

To assess the effect of the down-regulation of HOTAIR in CD117^+^CD44^+^-shHOTAIR on tumor metastasis, we used H&E staining to detect if there was tumor cell metastasis in the lung tissues. Compared with the mice injected with CD117^+^CD44^+^-scramble, no tumor cell metastasis was found in the lung tissues of the nude mice 64 days after they were injected with the CD117^+^CD44^+^-shHOTAIR (Figure 5E). It is thus evident from the results that the tumor cell growth and metastasis were significantly inhibited in the mice injected with CD117^+^CD44^+^-shHOTAIR compared with the mice injected with CD117^+^CD44^+^-scramble.

## Discussion

EOC is the most lethal gynecological malignancy in the world and approximately 30% of cancer patients have died this disease; this emphasizes the need for improved early detection and effective treatment [34]. Evidence has indicated that CSCs are believed to be the ‘seed cell’ in cancer recurrences and metastases, however, treatments targeted at CSCs have remained to be developed and studied [35]. Increasing evidence supports that CSCs possess mesenchymal characteristics and EMT ability, and that the decreased change in lincRNA HOTAIR was associated with alterations in specific EMT markers concurrent with reduced migratory potential [6,10,36]. In this regard, our current study was designed to investigate whether human EOC tissues and SKOV3 CD117^+^CD44^+^CSCs expresse HOTAIR and whether the decreased change of HOTAIR in human EOC SKOV3 CD117^+^CD44^+^CSCs are closely associated with the tumorgeniesis and metastasis. Indeed, our data indicated that human EOC patients’ tumor tissues and SKOV3 CD117^+^CD44^+^ CSCs had significantly high expressions of HOTAIR compared with the SKOV3 tumor tissues and SKOV3 non-CD117^+^CD44^+^CSCs, and that the down-regulated HOTAIR expression in CD117^+^CD44^+^-shHOTAIR markedly reduced cellular metastasis and invation in vitro as well as the tumorgeniesis in mice. This efficacy in CD117^+^CD44^+^-shHOTAIR was refelected in a lower clonogenic potential, a lower metastatic and invasive potential, and a weeker ability to form tumors in the xenografted mice than in CD138^−^CD34^−^-scramble. Importantly, the tumor cell lung metastasis was inhibited in the mice injected with CD117^+^CD44^+^-shHOTAIR, whereas the metastatic tumor cells were found in the lungs of the mice injected with CD117^+^CD44^+^-scramble.

The evident inhibitory activities in the SKOV3 cells transfected with siHOTAIR were apparently associated with inhibited the HOTAIR expression, which resulted in the increase of the epithelium feature molecule of the E-cadherin expression and reduced mesenchymal feature molecule of the Vimentin expression. The knockdown of the HOTAIR expression in CD117^+^CD44^+^CSCs may involve the inhibition of EMT of CD117^+^CD44^+^CSCs and the reduction of cellular migration and invasion potential and tumorgeniesis ability in vitro and in vivo. Nevertheless, the knockdown of the HOTAIR expression mechanism in inhibiting the metastasis of the SKOV3 cells and CD117^+^CD44^+^CSCs warrants further investigation.

In conclusion, the above-mentioned finding from our study is the first proof for demonstrating the novel role of the dawn-regulated HOTAIR in EOC SKOV3 CD117^+^CD44^+^CSCs, for suggesting the inhibitory cellular EMT, and for decreasing decreased the migration and and tumorgeniesis potential of CD117^+^CD44^+^CSCs. HOTAIR can be a potential therapeutic target for treatment of EOC patients.
